# Pitfalls and Challenges in Oral Plasma Cell Mucositis: A Systematic Review

**DOI:** 10.3390/jcm11216550

**Published:** 2022-11-04

**Authors:** Noemi Coppola, Tiziana Cantile, Federica Canfora, Daniela Adamo, Paolo Bucci, Michele Davide Mignogna, Stefania Leuci

**Affiliations:** 1 Oral Medicine Unit, Department of Neuroscience, Reproductive and Odontostomatological Sciences, University of Naples Federico II, 80131 Naples, Italy; 2 Department of Medicine, Surgery and Dentistry, Scuola Medica Salernitana, 84121 Salerno, Italy; 3 Department of Public Health, Section of Hygiene, University of Naples Federico II, 80131 Naples, Italy

**Keywords:** plasma cell mucositis, plasma cell gingivitis, plasma cell, oral plasma cell mucositis

## Abstract

Plasma cell mucositis (PCM) is an unusual idiopathic disorder characterized by dense infiltrates of plasma cells in submucosa. Clinical phenotypes of oral plasma cell mucositis (o-PMC) are heterogenous. A systematic review has been conducted, aiming to synthesize the available evidence on o-PCM. Literature search, study design, and data analysis were performed following PRISMA guidelines. The SPIDER and the PICO tools were used to structure the research question. In all, 79 case reports and 19 case series on a total of 158 patients (85 females and 73 males; average age: 44.1 years) were identified. Among oral sites involved, gingiva (65.82%) was the most frequent site. The main clinical phenotype was erythema (99.37%). In relation to symptoms, pain (60.76%) was the most reported. On histological examination, all samples showed a dense inflammatory infiltration with predominant plasma cells. The treatment regimens of o-PCM were summarized in six groups: irritant removal; topical/systemic corticosteroids; topical/systemic immunosuppressants/immunomodulators; surgery and similar treatments; radiotherapy and chemotherapy; other therapies, such as antifungals, antibiotics, and antivirals drugs. This is the first systematic review aimed to synthesize the findings of studies on o-PCM. The lack of universally shared information on etiological factors and the absence of international consensus of pharmacological protocols make o-PCM a diagnostic and therapeutic challenge.

## 1. Introduction

Plasma cell mucositis (PCM) is an unusual idiopathic disorder, histologically characterized by dense infiltrates of plasma cells in submucosa [1]. PCM was first reported by Zoon in 1952 as affecting the glans penis, and it was designated as Zoon’s balanitis [2,3].

Since then, similar pathologic changes have been seen at various mucosal sites, such as vulva, oral cavity (lips, buccal mucosa, palate, gingiva, tongue), epiglottis, larynx, pharynx, lower respiratory tract, conjunctivae, and skin [4].

Several nomenclatures have been used in the past to describe these clinicopathologic features: plasma cell orificial mucositis, idiopathic plasmacytosis, oral papillary plasmacytosis, and mucous membrane plasmacytosis [3]. Since cases reported in literature have been clinically and histopathologically indistinguishable from each other, the term oral plasma cell mucositis (o-PCM) has been proposed to facilitate the documentation of such disorders with oral involvement [5]. O-PCM is generally considered an extremely rare benign condition, which shows favorable prognosis, usually more common in males in the elderly age group [4,5].

Frequently, patients affected by o-PMC have a previous history of autoimmune or immunologically mediated diseases, such as Sjögren syndrome, autoimmune hepatitis, polymyositis, and diabetes mellitus. Nevertheless, these conditions are not present in all cases, and no single disease has been reported to be consistently associated [3,6].

O-PCM is commonly considered idiopathic, although hypersensitive reactions to certain types of antigens (chewing gum components, toothpaste, khat, or specific foods) have been proposed as possible etiologic factors [7].

Clinical phenotypes of o-PCM are heterogeneous, but, characteristically, patients show florid erythematous oral mucosa with cobblestone, nodular, papillomatous, granular, or velvety surface changes [6]. They can be asymptomatic or, more usually, present oral pain/burning sensation, gingivitis, sore throat, dry cough, and persistent hoarseness [4].

Secondary complications such as subglottic stricture, stenosis, and respiratory obstructions have been reported [5] as a consequence of fibrosis due to the healing of subepithelial damage.

O-PCM clinical features could mimic a wide range of entities, such as autoimmune mucocutaneous bullous diseases (AMBDs), lichen planus, candidiasis, contact mucositis, chronic glaucomatous sarcoidosis, systemic lupus erythematosus, Wegener’s granulomatosis, and squamous cell carcinoma, most of which can be ruled out with histological examination [8].

Plasma cell proliferation can also be correlated with some infectious diseases, such as syphilis, Castleman’s disease, a primary infectious disease of the lymph node, and, in recent times, COVID-19 [7].

The principal histological feature of o-PCM is a dense polyclonal plasmacytic infiltrate in the superficial lamina propria [6]. Immunohistochemical demonstration of polyclonality with no kappa or lambda light chain restriction allows the differentiation of o-PCM from myeloma, lymphoma, and extramedullary plasmacytoma [8]. The epithelium has been described as psoriasiform or showing pseudo-epitheliomatous hyperplasia, suprapapillary thinning, and dyskeratosis [9]. Few polymorphonuclear leucocytes and lymphocytes, with exocytosis and microabscess formation, as a result of ulceration and secondary non-specific inflammation can be also present. Russell bodies may be seen [8].

Therapeutic management of o-PCM is based mainly on relieving symptoms. Moreover, there is no international consensus about drug classes and regimens used to treat this disease [1]. Many treatments have been described, including topical, systemic, and intralesional corticosteroids [5]. Although steroid therapy is considered beneficial, resulting in disease stabilization and/or complete regression, adverse side effects prevent a prolonged use [6]. Antibiotics and antifungals have also been utilized [5]. Immunosuppressive drugs (methotrexate, tacrolimus, dapsone, mycophenolate mofetil, cyclosporine, colchicine, azathioprine infliximab, golimumab, and adalimumab) have been used, showing variable clinical success [6]. Systemic chemotherapy (cyclophosphamide, vincristine, and prednisolone) and low dose radiotherapy have also been reported in severe forms [4]. Furthermore, other strategies such as electrocoagulation, surgical excision, carbon dioxide laser, and cryotherapy have also been described [4].

However, in most of the reported cases, treatment resulted in stabilization of disease without a complete remission [1].

Although o-PCM was first described 70 years ago, it is rarely reported in dental literature, but it is important that PCM is recognized by head and neck practitioners, being its diagnosis dependent on clinical and histopathological correlation [5].

Actually, because of the inadequate clinical history and characterization of the disease that is mostly due to the lack of knowledge on etiology and pathophysiological mechanisms, practitioners’ decisions are usually based on clinical and histopathological data reported on few published case reports/case series [7]. Furthermore, to date, there has been no systematic review or meta-analysis on o-PCM in scientific literature. In order to bridge this gap, a systematic review has been conducted here, aiming at synthesizing all the available evidence on o-PCM, focusing on: patient characteristics (age/gender), symptoms, site/description of the lesion, comorbidities, histopathological and immunohistochemical examination, and therapeutic treatments.

## 2. Materials and Methods

Literature search, study design, and data analysis were performed following PRISMA (Preferred Reporting Items for Systematic reviews and Meta-Analyses) guidelines (Supplementary Materials). The SPIDER (Sample, Phenomenon of Interest, Design, Evaluation, and Research Type) tool was used to structure the research question: “What are the clinical, symptomatologic, histopathological, and therapeutic features of patients with o-PCM?”

### 2.1. Inclusion and Exclusion Criteria

All the studies that meet the following criteria were included: (1) Qualitative, quantitative, and mixed-method studies written in the English language; (2) Target population affected by o-PCM; (3) Studies provided applicable information on the clinical features, symptoms, histopathological characteristics, and treatment; (4) Reviews, case series, case reports, or letter to editor; (5) Region was non-limited. Articles that were not published in English or where the full text could not be acquired were excluded from the search. Opinion-based studies were not included (Table 1).

### 2.2. Search Strategy

A systematic search of qualitative literature from any year published up to January 2022 was conducted in electronic databases of PubMed and Scopus. Furthermore, the reference lists of retrieved articles were reviewed to identify potential relevant studies. The search was performed using a combination of the following medical subject heading terms: “plasma cell”, “mucositis”, “oral”, “orificial”, “ILPMD”, “balanitis”, “plasmacytosis”, “mucositis-dermatitis”, “gingivitis”, “oral papillary plasmacytosis”, and “mucous membrane plasmacytosis”. The elected search terms were combined with Boolean operators for detailed electronic search: “plasma cell” AND “mucositis”; “plasma cell” AND “mucositis” AND “oral”; “plasma cell” AND “mucositis” AND “orificial”; “plasma cell” AND “balanitis”; “plasma cell” AND “mucositis-dermatitis”; “plasma cell” AND “gingivitis”; “ILPMD”; “ILPMD” AND “oral”; “oral” AND “plasmacytosis”;“orificial” AND “ILPMD”;“orificial” AND “plasmacytosis”; “idiopathic plasmacytosis”; “idiopathic plasmacytosis” AND “oral”; “idiopathic plasmacytosis” AND “orificial”; “oral” AND “papillary plasmacytosis”; “orificial” AND “papillary plasmacytosis”; and “mucous membrane” AND “plasmacytosis”.

### 2.3. Study Selection

Two researchers independently screened the titles and abstracts to identify eligible records. Then, in line with inclusion and exclusion criteria, a full-text eligibility assessment was performed by the two reviewers in a blinded process, after which the process of referencing and citation searching was made. Disagreements were resolved by discussion and consensus.

### 2.4. Data Extraction and Quality Assessment

A standardized form was used to extract data from the included studies. To meet the purpose of the review, two reviewers independently collected the following data: author’s name, year of publication, age, gender, comorbidities, symptoms, site of the lesions, clinical features, histopathology (direct immunofluorescence and/or immunohistochemistry, if available), and therapeutical management. When a study included a group of patients whose age was defined by a range, the mean value between the major and minor age of the range was used, and the mean value was calculated respecting the numerical weight of the patients. The quality of studies, according to types of studies, was assessed using the CARE checklist. The CARE guideline consists of 13 sections with 30 sub-items. Adherence to each sub-element was assessed on a dichotomous basis (yes/no).

### 2.5. Synthesis of Results

Descriptive statistics were performed for the examined variables. All categorical variables (i.e., gender, comorbidities, symptoms, site of the lesions, clinical features, histopathology, and therapeutical management) were reported as counts and percentages. Age, being a continuous variable, was reported as mean.

A statistical software (IBM SPSS Statistics v.25, IBM Inc., Armonk, NY, USA) was used for calculations.

## 3. Results

### 3.1. Study Selection

**Figure 1:**
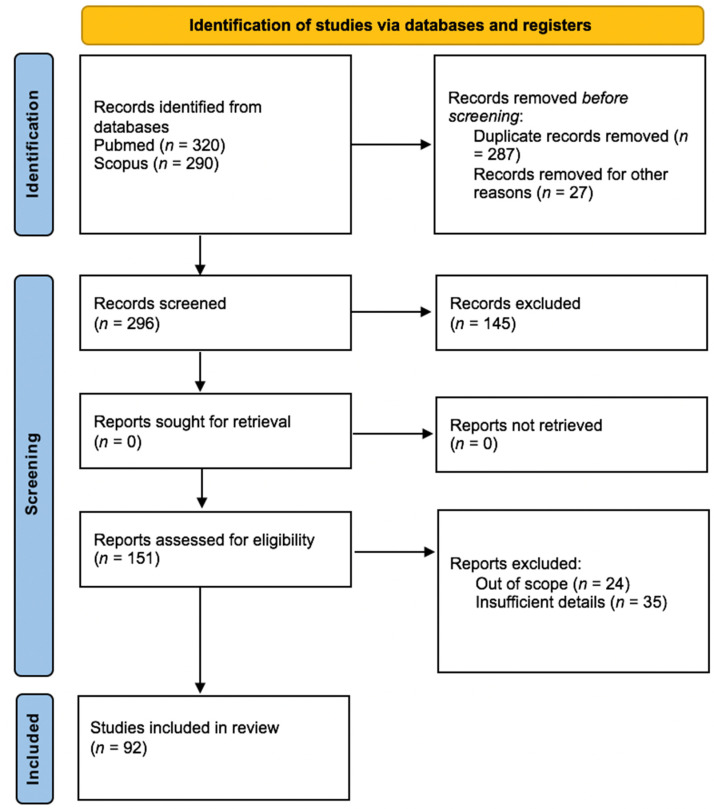
PRISMA 2020 flow diagram.

The flow diagram (Figure 1) depicts the results of the literature search and the study selection process.

The electronic searches retrieved 296 records after removing duplicates. After reviewing the titles and abstracts, we examined the full text of the remaining 151 citations, of which 92 fulfill inclusion criteria.

### 3.2. Study Characteristics and Results of Data Synthesis

Within this group, we identified 73 (79.35%) case reports and 19 (20.65%) case series on a total of 158 patients. Eighty-five patients were women, seventy-three were men, and the average age of the patients was 44.1 years. In 43 (27.21%) patients, an intraoral irritant, such as chewing gum and toothpaste, was identified (Table 2). The data extracted from the included articles are presented in Table S1.

### 3.3. Clinical Manifestations

**Figure 2:**
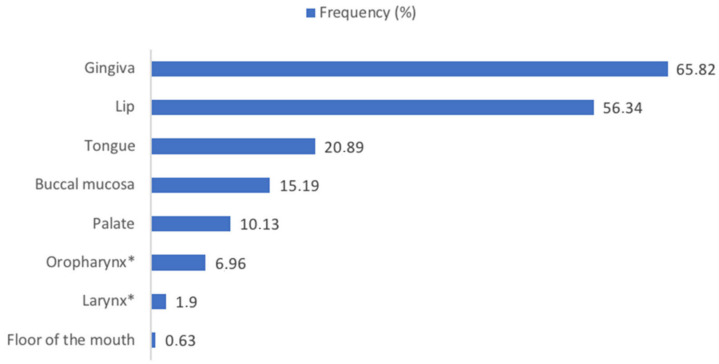
Frequency of the oral sites involved by o-PCM. * Percentage of cases in which a pharyngeal and laryngeal extension beyond the oral one is reported.

Figure 2 shows the frequency of the oral sites involved by o-PCM. The percentages exceed 100 because most of the patients showed multiple site involvement. The gingiva (65.82%) was found to be the most frequent site in this cohort; the most uncommon site reported was the floor of the mouth (0.63%).

Of the 158 reported cases, the main clinical phenotype was erythema (99.37%) followed by bleeding (46.20%) and swelling (39.87%). Edema, erosions, ulcerations, and gingival hypertrophy were also described. The clinical manifestations by anatomical site are summarized in Table 3.

**Figure 3:**
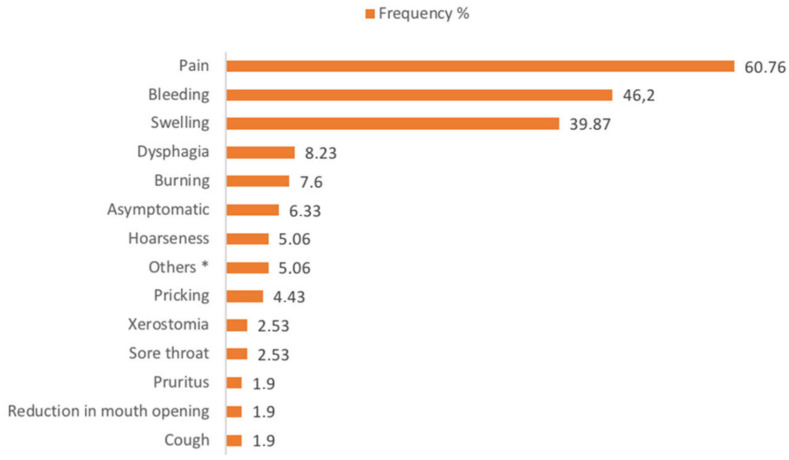
Frequency of oral symptoms and signs. * Globus pharyngeus: 2 (1.27%); Dysphonia: 2 (1.27%); Bilateral cervical lymphadenopathy: 2 (1.27%); Dysgeusia: 1 (0.63%); Asthenia 1 (0.63%); Aesthetic deformities: 1 (0.63%).

The frequency of oral symptoms and signs was reported in Figure 3, where pain (60.76%) and bleeding (46.20%) were the most reported.

### 3.4. Histopathological Findings

On histological examination, all samples showed a dense inflammatory infiltration with predominant plasma cells. In some cases, analysis of the literature data revealed a mixed infiltrate with the following frequency: 29 (18.35%) with lymphomonocytes, 16 (10.13%) with neutrophils, 15 (9.49%) with eosinophils, 9 (5.70%) with mast cells, and 4 (2.53%) with macrophages. Another common histological feature was epithelial hyperplasia (26.58%). A comprehensive report on histopathological features is shown in Table 4.

Kappa and lambda immunohistochemistry (IHC) was performed in 44 samples (27.85%). Of these, a normal ratio of kappa and lambda expressing cells was reported in 26 (59.09%) patients, a predominance of kappa and lambda light chain expression is identified in 17 (38.64%) and 1 (2.27%) samples, respectively.

### 3.5. Treatment Regimens

**Figure 4:**
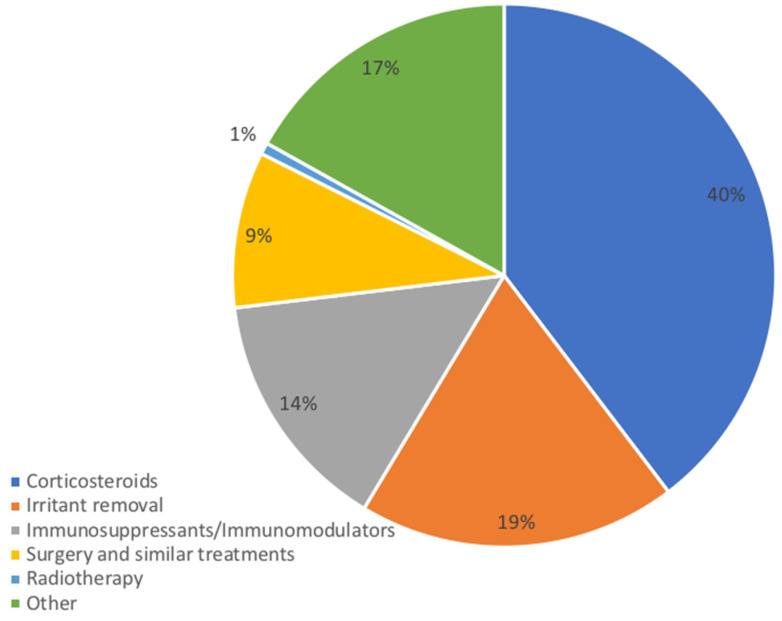
Therapeutic strategies for o-PCM.

The treatment regimens of o-PCM in the included studies showed a great variability among the different authors. From the research carried out, it was possible to identify six groups of therapies (Figure 4):

1. Irritant removal

The identification and attempted removal of the irritant was observed in 55 cases (18.96%), of which there were 29 cases (52.73%) with rapid positive response and complete clinical remission, 16 (29.09%) with partial response, and 10 (18.18%) that did not show any clinical improvement. In cases of partial response or no response, additional therapeutic strategies were associated: non-surgical periodontal therapy in eight cases (30.77%) with gingival involvement, surgery in eight cases (30.77%), corticosteroids in eight cases (30.77%), and systemic antibiotics in two cases (7.69%).

2. Topical and systemic corticosteroids

Among the 290 treatment regimens described, 115 (39.66%) were corticosteroids. The molecules administered and the efficacy of the steroid therapy are shown in Table 5. In the 19 cases (16.52%) in which corticosteroids were used effectively in association with other therapy, the molecule administered in combination was always found to belong to the class of corticosteroids in 13 cases (68.42%), removal of the identified irritant was associated in 13 cases (68.42%), an immunosuppressant (Mycophenolate Mofetil and Tacrolimus) was associated in 2 cases (10.53%), and, in 2 cases (10.53%), the association with phototherapy, topical antifungals, and antihistamines was reported.

3. Topical and systemic immunosuppressants/immunomodulators

Out of 290 types of treatment and molecules administered, 42 (14.48%) belong to the category of immunosuppressants/immunomodulators. The molecules administered and the efficacy of the immunosuppressants/immunomodulators are showed in Table 4. In the eight cases (19.05%) in which the Immunosuppressants/Immunomodulators were used effectively in association with another therapy, the molecule administered in combination was found to belong to the class of corticosteroids in five cases (62.5%), phototherapy was associated in one case (12.5%), ketoconazole was associated (12.5) in one case (12.5%), a corticosteroid in one case (12.5%), and an antihistamine in one case (12.5%).

4. Surgery and similar treatments

Out of 290 types of treatment and molecules administered, 27 (9.31%) belong to the category of surgical or similar treatments. The effectiveness of therapy with surgery and the like in all patients is described in Table 5. In five cases (18.51%) traditional surgery was used effectively in association with other therapy: one case (20%) in association with removal of the irritant, two cases (40%) in association with etiological periodontal treatment, one case (20%) in association with removal of the irritant + periodontal etiological therapy, and one case (20%) in association with levocetirizine (5 mg/day) and triamcinolone acetonide 0.1%.

5. Radiotherapy

Out of 290 types of treatment and molecules administered, 2 (0.69%) were radiotherapy protocols (3600 rads/16 fractions, 25 Gy/14 fractions). Among these patients, complete clinical remission was achieved only in one case through association with Radiotherapy, Prednisolone, and Surgery.

6. Other therapies

Out of 290 types of treatment and molecules administered, 49 (16.90%) consisted of different therapies such as antibiotics, antifungals, antivirals, and antihistamines. In particular, the molecules administered and their therapeutic efficacy are shown in in Table 4. In six cases (12.24%) antibiotics, antifungals, antivirals, and antihistamines were used effectively in association with other therapy, in five cases (83.33%) the molecule administered in combination was found to belong to corticosteroids, and in one case (16.67%) removal of the identified irritant was associated.

## 4. Discussion

Although the first case of o-PCM was reported in the literature in 1960 [10], most of the cases were published after 2000 (22 papers before 2000 vs. 70 papers after 2000). This increase could be due both to greater attention by clinicians and patients to alterations of the oral mucosa and to an increase in exogenous stimuli to which the oral cavity is exposed with greater antigenic complexity. There is no gender prevalence among the patients included in this review, unlike other oral diseases, such as oral lichen planus, with exclusively immune-mediated pathogenesis [11].

From the literature analysis, it emerged that there is not enough data to define the etiology of the disease. However, it was possible to identify three etiological hypotheses: allergic process, inflammatory disease, and idiopathic manifestation [12]. The first category includes o-PCMs that develop following sensitization to an antigen. In the cases included in this review, an allergen was identified in 27.21% of patients. The etiological factors that act as local irritants identified with some recurrence are chewing gum and herbal toothpastes.

The second etiological category identified by Gargiulo et al. is the inflammatory class. Events such as trauma or parafunctional behaviors are sometimes the cause of the induction of plasma cell sub-epithelial infiltrates [13,14]. Moreover, the evidence of plasma cell-related inflammatory lesions in areas of contact and rubbing in anatomical districts other than the oral cavity (genital, axillary, skin) supports the hypothesis of trauma and micro-trauma as a possible etiology of the plasma cell infiltrate [15]. Additionally, exposure to fungal (candida albicans), viral (herpes virus), or bacterial (dental plaque) infections can somehow act as a trigger for the development of a plasma cell infiltrate [16,17]. Particularly, Candida Albicans infection is often cited and reported in the literature as it appears to be related to a delayed hypersensitivity reaction, such as to induce the accumulation of plasma cell infiltrate indirectly [17,18]. A single case is also reported in which chronic exposure to fungal hyphae may have led to a slight but chronic damage to the integrity of the mucosal surface such as to induce plasma cell lesions, even in the absence of clinically appreciable colonization [19]. A role of bacterial plaque has also been hypothesized, with many documented cases of development of plasma cell lesions in plaque-related anatomical areas, in some cases also with periodontal disease in association with o-PCM [7,20,21]. It is necessary to emphasize that the presence of a band-like plasma cell infiltrate is found in plaque-related gingival hyperplasia and chronic periodontitis, which, therefore, fall within the differential diagnosis. In this case, the condition should regress following the removal of the irritating etiological agent. However, plaque-induced gingivitis would only involve the marginal gingiva and not the entire height of the adherent gingiva as usually happens in cases of o-PCM [7]. Moreover, the plaque-induced gingivitis would not be extremely resistant to periodontal etiological treatment, as can happen in cases of gingival o-PCM [22]. It is likely, in these cases, that there may be a multifactorial etiology in which the presence of bacterial plaque is a pejorative of an underlying pathological condition.

Although the pathogenetic mechanism that induces the development of o-PCM is currently unknown, in the literature the analysis has focused on the development and differentiation of B cells (and the relationship with T-cells and macrophages) and on the role of the pro-inflammatory cytokines [14]. The good response to fusidic acid therapy reported in the literature supports the role of inflammatory cytokines in the onset of o-PCM [23]. In fact, fusidic acid, in addition to the antibacterial activity, can suppress the production of inflammatory cytokines such as IL-2, interferon-gamma, IL-1, and IL-6 [24]. Finally, in some cases, given the frequent association between aggressive periodontitis with bone resorption and o-PCM, a synergy between pro-inflammatory cytokines of both lesions has been hypothesized, which exacerbates the clinical manifestation. According to Baughman et al. following an experiment of induction of plasma cell infiltrates by means of allergic/irritant stimuli, the pathogenetic mechanism leading to o-PCM would not be represented by a specific response to a stimulus but would be an early response of the immune system to unidentified stimuli [25]. Ultimately, it is not possible to identify a single antigen, chemical mediator, or cell that may be responsible for the induction of the plasma cell infiltrate. It is likely that chronic inflammation leads to mechanisms of loco-regional immunological dysregulation and induction of plasma cell migration.

The studies included showed that the most common affected site by o-PCM was gingiva. Oral mucosa is a gateway into the human body, and it is constantly exposed a plethora of stimuli, including food, allergens, microbes, and mechanic stimuli [26]. Gingiva is a particularly vulnerable site due to her histological features. In fact, recent studies based on flow cytometric analysis of oral mucosa in healthy subjects highlighted a more intense infiltrate of inflammatory cells in the gingiva than in the other intraoral mucosal sites [27]. Neutrophils, other granulocytes, monocular phagocytes, and lymphocytes are the most common inflammatory cells described [26]. These data are consistent with increased trigger exposure at this site, and it could be the reason why the gingiva is the most involved oral site.

Clinically, the o-PCM is very polymorphic and heterogeneous. To regard the phenotype of the disease, the main feature was erythema that involved the totality of the patients. In addition to erythema, the review of the literature revealed a greater variability of morphological aspects ranging from lesions characterized by loss of substance, such as ulcerations and erosions, to lesions with an increase in the epithelial-mesenchymal component, such as gingival hypertrophy and wart-like lesions. The multiplicity of the clinical manifestations does not make it possible to define a pathognomonic aspect of o-PCM. When there is gingival involvement, clinical manifestations may extend beyond the mucogingival junction; in the involvement of the lips, there were erythema in the labial semi-mucosa and crusted-hemorrhagic lesions in the cutaneous side [3,7,28,29]. Moreover, in 2021 for the first time, a pure bullous phenotype was described in 25 patients, mimicking many blistering diseases [7]. In these cases, a careful differential diagnosis with other bullous oral mucositis is necessary, for example the pemphigus and pemphigoid group, including in the diagnostic pathway ELISA tests for the available antigens and direct immunofluorescence.

In relation to clinical heterogeneity, the symptoms are highly variable. The most common one was pain, where there is loss of substance, and sometimes bleeding. From the analysis of the data reported in the literature, only 6.33% of patients were asymptomatic. In cases where pharyngeal or laryngeal extension of the disease is described, patients complained of pharyngeal globus, cough, and hoarseness. In most of the examined papers, the correspondence between clinical manifestation, site of the lesion, and symptoms was not indicated. For this reason, it was not possible to make a correlation analysis between the three variables. So, the overall frequency of reported symptoms is neither site-specific nor related to clinical manifestation.

Histological findings showed a dense plasma cell-rich infiltrate in all specimens, confirming the diagnosis of o-PCM. In most of the samples, the plasma cells were localized in lamina propria; other times the plasma cell infiltrate slightly involved the epithelium and could be separated by septa and bundles of fibrous connective tissue [28,30,31]. Plasma cells are not atypical or anaplastic, and prominent nucleoli are not described. Although plasma cells were the most represented share, from the analysis of the literature there emerged cases with polymorphic infiltrate with the presence of lymphocytes, neutrophils, and eosinophils in variable percentages. Furthermore, microscopic analysis often revealed epithelial hyperplasia, described as papillary hyperplasia, pseudoepitheliomatous hyperplasia, or psoriasiform hyperplasia. From literature data, in patients with o-PCM, the epithelium can be the site of atrophy, edema, spongiosis, acanthosis, and micro-abscesses. Immunohistochemistry of Kappa and lambda was performed only in a low percentage of patients, showing, in most cases, a normal ratio of Kappa and lambda chains and a predominance of kappa light chains in 17 patients. Immunohistochemistry in o-PCMs should always be performed to confirm the polyclonality of the Igs and, thus, confirm that the lesion is a manifestation of a non-neoplastic polyclonal benign reactive process. It is, therefore, necessary to exclude restrictions for light or heavy chains and expression of oncological disease. The execution of immunohistochemistry only in a limited number of samples is data to be analyzed as it could hide a lack of attention to a probable systemic involvement during o-PCM.

**Figure 5:**
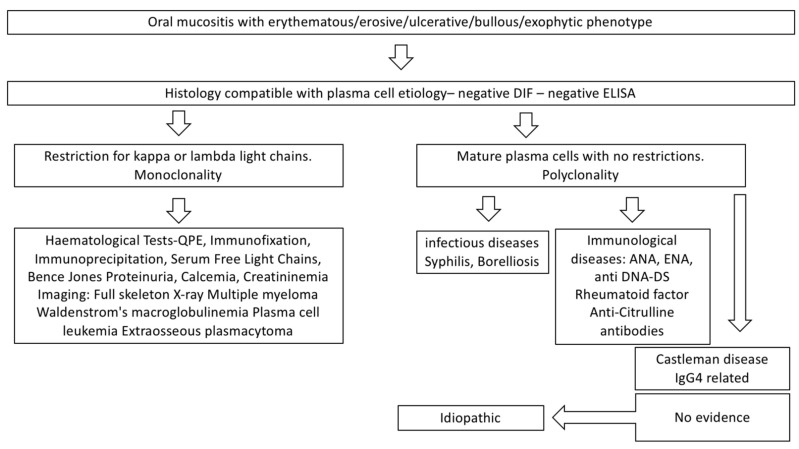
Diagnostic pathway for o-PCM.

The diagnosis of o-PCM makes use of anamnestic collection, physical examination, serological tests, and histological examination. A proposal diagnostic pathway is shown in Figure 5.

The lack of an international consensus on what the therapeutic “gold standard” is reflects the clinical and symptomatological heterogeneity. The various therapeutic strategies used over time have given very different outcomes, at times satisfactory with complete clinical remission, at other times unsatisfactory and not resolving, characterized by partial clinical remission or by frequent relapses of the disease or absence of clinical response. The identification and removal of any local irritant is the best therapeutic strategy, with a success of up to 52% of cases managed in this way. Identification of the potential irritant (herbal toothpaste, chewing-gum, spicy foods, or dental bacterial plaque) during anamnestic collection represents the first essential step of the therapeutic strategy. As indicated in numerous cases, when the etiological factor is identified, the treatment consists of the removal of the same, which is often followed by resolution [18,20,32,33]. However, this does not always occur and, therefore, it is possible to resort to the use of surgical or pharmacological strategies at a later stage [17,34]. Corticosteroids are among the most widely used pharmacological protocols, although their immediate and long-term efficacy is uncertain. There is various evidence supporting the therapeutic efficacy with complete clinical resolution of very extensive lesions [15,35]. The most used molecules are Triamcinolone Acetonide, Prednisolone, Prednisone, and Betamethasone. Sometimes corticosteroids are only effective immediately, failing to guarantee therapeutic success over time; when their use is stopped, relapse of the disease occurs suddenly [34,36]. Systemic steroid therapy appears to have good efficacy, although it may be burdened with greater onset of dose-related side effects. The effectiveness of topical steroid therapy, unfortunately, depends on the thickness of the epidermal barrier and the amount of plasma cell infiltrate. Hence, there is sometimes a poor response to topical treatments [30,37]. A well-known modality that enhances the effects of topical corticosteroid therapy is intra- or peri-lesional infiltration [37]. The efficacy of immunosuppressants/immunomodulators is also relatively low, 23.81% in monotherapy and 19.05% in combination. No response to treatment was reported in 57.14% of cases. The molecule in this class of drugs that has given the best results is Tacrolimus, whose mechanism of action consists of inhibiting the transcription of cytokine genes, including interleukin IL-3, IL-4, IL-5, and TNF. Particular consideration is given to the role of non-surgical periodontal therapy in association with further treatments in the control and clinical remission of gingival lesions. In fact, the muco-bacterial plaque induces and supports a localized chronic inflammatory state that allows the perpetuation of cell-mediated and, consequently, cytokine-mediated immunological activation that do not to allow a restitutio ad integrum of the epithelium, even in the presence of a concomitant systemic pharmacological protocol.

Although the bacterial pathways are not yet well understood, it is assumed that some bacterial species, such as *P. Gingivalis*, *Staphylococcus epidermidis*, *Enterococci* spp., viruses, and Candida may delay the healing of ulcerative lesions [38,39].

To date, there is no evidence in the literature that directly causally correlates microbial species and oral mucositis. Oral dysbiosis are, therefore, likely to act as co-factors, increasing the morbidity and healing time of the lesions. From what emerges from the literature, it is highlighted that therapeutic management is the real challenge of o-PCM.

Finally, it should be remembered that plasma cell infiltrate in an oral biopsy can be an expression of underlying systemic diseases. Therefore, as shown in Figure 5, it is necessary to exclude any other disease whose oral histological manifestation involves the presence of plasma cells: among these we remember monoclonal gammopathy of undetermined significance, multiple myeloma, lymphoplasmacytic lymphoma/Waldenstrom macroglobulinemia, and amyloidosis [40].

Moreover, in the future, it could be interesting to evaluate whether the pain that turns out to be the main symptom is really correlated to the clinical manifestation. Clinical findings of o-PCM are heterogeneous and, like oral lichen planus, the o-PCM recognizes an immune-mediated pathogenesis with inflammatory infiltrate, although the cell populations detected in the infiltrate are different in the two pathologies. It has recently been demonstrated that, in oral lichen planus, there is a poor correspondence between the site of lesions and the site of the symptoms, and, to date, it is not possible to evaluate this aspect in o-PCM [41].

The present study had certain limitations that should be addressed. First, due to the lack of observational studies, randomized controlled trials, and meta-analyses on o-PCM, it has been necessary to include case reports and case series in the present review. Second, in consideration of the typology of articles selected for the review, it was not possible to follow all the points of the PRISMA checklist. Furthermore, the research protocol has not been registered on any of the current databases for systematic reviews (i.e., International Prospecting Register of Systematic Reviews (PROSPERO) or the Systematic Review Register of the Joanna Biggs Institute (JBI).

## 5. Conclusions

Although it was possible to identify three etiological hypotheses responsible for o-PMC (allergic process, inflammatory disease, and idiopathic manifestation), the pathophysiological mechanism is still not clearly understood, and there is no international consensus about drug classes and therapeutic regimens used to treat this disease. Furthermore, o-PCM is rarely reported in dental literature, but it is essential to improve awareness of this disease among dentists, oral surgeons, and oral medicine specialists to obtain a timely diagnosis and begin appropriate therapy.

This is the first systematic review aimed to synthesize the findings of studies on o-PCM. The lack of universally shared information on etiological factors, together with the morphological non-specificity of oral lesions, and the absence of international consensus of pharmacological protocols make o-PCM a diagnostic and therapeutic challenge.

Therefore, in the future, properly defined randomized trials with large sample sizes are needed to bridge the gap on etiopathogenesis knowledge and to allow a more effective clinical management of o-PCM.

## Figures and Tables

**Table 1:** Inclusion and exclusion criteria.

	Inclusion Criteria	Exclusion Criteria
Language	English	All other languages
Sample (S)	Patients affected by o-PCM	Patients who do not possess the inclusion criteria for this study
Phenomenon of interest (PI)	Histopathological features of o-PCM	
Design of study (D)	Case series, case reports, reviews, letter to editor	Opinion-based studies
Evaluation (E)	Clinical features, symptoms, histopathological characteristics, treatment of o-PCM	Studies that do not report even just a component between clinical, symptomatological, histopathological and/or DIF and/or immunohistochemical characteristics, therapy
Research type (R)	Qualitative, quantitative, and mixed-method studies	-

**Table 2:** Type of contact irritant.

	Frequency N (%)
Chewing-gum	18 (41.86%)
Toothpaste	15 (34.88%) 12 herbal toothpastes 2 cinnamon-flavored toothpastes 1 sodium lauryl sulphate toothpaste
Khat	7 (16.28%)
Foods	4 (9.30%) 1 *Capsicum annuum* L. 1 Colocasia 1 Spices 1 Mint-flavored candy
Other	1 (2.33%) Syzygium aromaticum

**Table 3:** Distribution of clinical manifestations of o-PCM by anatomical site.

Clinical Features	Palate N° (%)	Gingiva N° (%)	Tongue N° (%)	Buccal Mucosa N° (%)	Lip N° (%)	Oropharynx N° (%)	Larynx N° (%)	Floor of the Mouth N° (%)	Total Patients N° (%)
Erythema	11 (7.01)	79 (50.32)	20 (12.74)	15 (9.55)	23 (14.65)	5 (3.19)	3 (1.91)	1 (0.64)	157 (99.37)
Swelling		21 (42)	2 (4)	1 (2)	25 (50)		1 (2)		50 (31.65)
Edema	3 (6.12)	38 (77.55)	1 (20.04)	2 (4.08)	1 (2.04)	4 (8.16)			49 (31.01)
Erosion	2 (5.13)	2 (5.13)	2 (5.13)	5 (12.82)	27 (69.23)	1 (2.56)			39 (24.68)
Ulceration	2 (5.88)	6 (17.65)	3 (8.82)	7 (20.59)	15 (44.12)	1 (2.94)			34 (21.52)
Angular cheilitis									30 (18.99)
Secondary impetigo					20 (100)				20 (12.66)
Gingival hypertrophy									17 (10.76)
Fissuration			7 (41.18)		10 (58.82)				17 (10.76)
Erythematous plaques	1 (8.33)	1 (8.33)	1 (8.33)		8 (66.67)	1 (8.33)			12 (7.60)
Bone loss									11 (6.96)
Pseudo-pockets									11 (6.96)
Desquamative gingivitis									7 (4.43)
Cobblestone appearance	3 (42.86)	1 (14.29)		2 (28.57)		1 (14.29)			7 (4.43)
Warty lesion	1 (16.67)		1 (16.67)	1 (16.67)	3 (50)				6 (3.80)
Keratotic plaques					5 (100)				5 (3.16)
Hyperplasia		4 (80)		1 (20)					5 (3.16)
Papillary hyperplasia	3 (60)					2 (40)			5 (3.16)
Tongue Atrophy									2 (1.27)
Exophytic sessile lesion		1 (100)							1 (0.63)
Exophytic pedunculated lesion		1 (100)							1 (0.63)
Small pink papules					1 (100)				1 (0.63)

**Table 4:** Summary of histopathological findings.

Histopatological Features	Frequency N (%)
*Sub-epithelial features*	
Dense plasma cell-rich infiltrate	158 (100)
Lymphocytes	29 (18.35)
Dilated capillaries	21 (13.29)
Neutrophils	16 (10.13)
Eosinophils	15 (9.49)
IgG	10 (6.33)
Mast cells	9 (5.70)
Russel bodies	9 (5.70)
Micro-abscess	8 (5.06)
Macrophages	4 (2.53)
Fibrosis	1 (0.63)
*Epithelial features*	
Epithelial hyperplasia *	42 (26.58)
Parakeratosis	22 (13.92)
Edema	20 (12.66)
Acanthosis	19 (12.02)
Atrophy	12 (7.60)
Spongiosis	11 (6.96)
Exocytosis	10 (6.33)
Elongated rete pegs	10 (6.33)
Vacuolar degeneration	8 (5.06)
Acantholysis	1 (0.63)

* 7 (4.43%) papillary hyperplasia; 6 (3.80%) pseudoepitheliomatous hyperplasia; 5 (3.16%) psoriasiform hyperplasia.

**Table 5:** Summary of therapeutic regimens reported in literature.

	Efficacy (N)	Efficacy in Association with Other Therapy (N)	Partial or Null Effect (N)	Total
Corticosteroids				
Prednisone	9	3	3	15
Triamcinolone Acetonide	8	3	7	18
Prednisolone	4	4	8	16
Betametasone	4	1	4	9
Clobetasol	6	0	2	8
Fluocinolone	1	1	5	7
Methylprednisolone	1	0	7	8
Hydrocortisone	0	0	3	3
Unspecified	0	7	24	31
Total	33 (28.70%)	19 (16.52%)	63 (54.78%)	
Immunosuppressants/Immunomodulators				
Tacrolimus	8	5	12	25
Pimecrolimus	1	0	2	3
Mycophenolate Mofetil	0	1	2	3
Methotrexate	0	0	2	2
Colchicine	0	0	2	2
Azathioprine	0	0	2	2
Cyclosporine	1	0	0	1
Levamisolone	0	2	0	2
Etretinato	0	0	1	1
Hydroxychloroquine	0	0	1	1
Total	10 (23.81%)	8 (19.05%)	24 (57.14%)	
Surgery and similar treatments				
Traditional surgery	9	5	6	20
Laser surgery	2	0	0	2
Cryotherapy	1	0	2	3
Electrocoagulation	1	0	0	1
Phototherapy	1	0	0	1
Total	14 (51.85%)	5 (18.51%)	8 (29.63%)	
Other therapies				
Antifungals	3	2	10	15
Fusidic acid	2	0	3	5
Chlorphenamine maleate	1	0	0	1
Antibiotics, unspecified	0	2	13	15
Antivirals, unspecified	0	0	1	1
Acyclovir	0	0	2	2
Valacyclovir	0	0	1	1
Unspecified antihistamines	0	0	1	1
Diphenhydramine	0	0	1	1
Promethazine	0	1	2	3
Dapsone	0	1	3	4
Total	6 (12.24%)	6 (12.24%)	37 (75.51%)	

## Data Availability

The data that support the findings of this study are available from the corresponding author upon reasonable request.

## Supplementary Materials

The following supporting information can be downloaded at: https://www.mdpi.com/article/10.3390/jcm11216550/s1, Table S1: Oral plasma cell mucositis: A review of literature.
