# Supplementary material for: Pitfalls and Challenges in Oral Plasma Cell Mucositis: A Systematic Review
Source: J Clin Med. 2022 Nov 4;11(21):6550. doi: 10.3390/jcm11216550 (PMC9659091; doi:10.3390/jcm11216550)
Supplement: Supplementary file 1 [file jcm-11-06550-s001.zip › jcm-1969636-supplementary.pdf]

Table S1. Oral plasma cell mucositis: a review of literature.

Study/year of publication	Age/Gender	Symptoms	Site of the lesion	Description of the lesion	Comorbidities	Histopathology	Immuno-histochemistry
Shanahan et al./ 2020	62/F	• Xerostomia<br>• Dysphagia<br>• Hoarseness<br>• Swelling	• Palate<br>• Buccal mucosa	• Erythema<br>• Edema<br>• Ulceration	• Allergic rhinitis<br>• Occasional migraines<br>• Gastro-oesophageal reflux disease	• Dense plasma cell-rich infiltrate	Normal k:λ ratio
Dhir et al./ 2012	28/M	• Swelling<br>• Burning<br>• Bleeding	• Gingiva	• Gingival hypertrophy<br>• Erythema<br>• Edema<br>• Pseudo-pockets	NA	• Spongiosis<br>• Psoriasiform epithelial hyperplasia<br>• Leukocytes exocytosis<br>• Neutrophilic micro-abscess	NA
Dos Santos et al./ 2019	56/M	• Pain	• Lip	• Ulceration<br>• Secondary impetigo	• Hypertension<br>• Diabetes mellitus	• Parakeratosis<br>• Dense plasma cell-rich infiltrate<br>• Lymphocytes	k:λ ratio= λ>k

						• Eosinophils<br>• Russell bodies	
Kuriyama et al./2019	65/M	• Pain<br>• Dysphagia	• Lip	• Ulceration	• Intellectual disability<br>• Epilepsy<br>• Benign prostatic hypertrophy	• Epithelial hyperplasia<br>• Dense plasma cell-rich infiltrate	NA
Liu et al. /2017	18/F	• Swelling<br>• Bleeding<br>• Pain	• Lip	• Erosion<br>• Secondary impetigo<br>• Swelling	NA	• Atrophy<br>• Dense plasma cell-rich infiltrate<br>• Lymphocytes<br>• Neutrophils<br>• IgG+	Normal k:λ ratio
Arun et al./2017	13/M	• Bleeding<br>• Swelling	• Lip Gingiva	• Erythema<br>• Pseudo-pockets<br>• Swelling	NA	• Dense plasma cell–rich infiltrate	k:λ ratio= k>λ
Panpradit and Lapthanasupkul et al./ 2017	35/F	• Bleeding<br>• Burning<br>• Swelling	• Gingiva<br>• Lip	• Erythema<br>• Edema<br>• Erosion	NA	• Epithelial hyperplasia<br>• Spongiosis<br>• Exocytosis	DIF: IgG- , IgM +, IgA+, C3+, Fibrinogen+.

				• Swelling		• Dense plasma cell-rich infiltrate<br>• Lymphocytes	
Lee JY et al./2017	64.7*/9xM, 4xF	• 7x Pricking<br>• 6x Pain<br>• 3x Pruritus<br>• 2x Burning<br>• 9x Bleeding<br>• 1x Asymptomatic<br>• 9x Swelling	• 13x Lip	• 8x Erythematous plaques<br>• 5x Keratotic plaques<br>• 10x Erosion<br>• 2x Ulceration<br>• 9x Swelling	NA	• 13x Dense plasma cell-rich infiltrate<br>• 9x Eosinophils<br>• 5x Neutrophils<br>• 8x Acanthosis<br>• 4x Edema<br>• 6x Parakeratosis<br>• 8x Vacuolar degeneration	x 9 k:λ ratio= k>λ
Vinay et al./2016	38/F	• Pain<br>• Bleeding<br>• Swelling	• Lip<br>• Gingiva	• Secondary impetigo<br>• Swelling<br>• Erosion	NA	• Dense plasma cell–rich infiltrate<br>• Lymphocytes	Normal k:λ ratio

Trehan et al./ 2016	39/M	• Pain Dysphagia	• Lip<br>• Buccal mucosa<br>• Gingiva	• Erythema<br>• Erosion<br>• Ulceration	• Iron-deficiency anaemia	• Dense plasma cell–rich infiltrate	NA
Prasanna et al./ 2016	19/F	• Swelling<br>• Bleeding	• Gingiva<br>• Lip	• Gingival hypertrophy<br>• Edema<br>• Erythema<br>• Swelling	NA	• Pseudo-epitheliomatous hyperplasia<br>• Parakeratosis<br>• Spongiosis<br>• Lymphocytes<br>• Neutrophilic micro-abscess<br>• Dense plasma cell–rich infiltrate<br>• Russel bodies<br>• Dilated capillaries	NA
	15/M	• Swelling<br>• Dysphonia<br>• Bleeding	• Gingiva<br>• Lip	• Gingival hypertrophy<br>• Swelling	• Sea food allergy	• Pseudo-epitheliomatous hyperplasia<br>• Parakeratosis<br>• Spongiosis	NA

						• Lymphocytes<br>• Neutrophilic micro-abscess<br>• Dense plasma cell–rich infiltrate<br>• Russel bodies<br>• Dilated capillaries	
Saluja et al./ 2016	25/M	• Dysphagia<br>• Swelling<br>• Bilateral cervical lymphadenopathy	• Gingiva	• Gingival hypertrophy<br>• Erythema<br>• Pseudo-pockets<br>• Bone loss<br>• Swelling	• Polyclonal gammopathy<br>• Aggressive periodontitis	• Edema<br>• Dense plasma cell–rich infiltrate<br>• Lymphocytes	NA
Yamaguchi et al./ 2016	58/M	• Pain	• Lip	• Erythema<br>• Ulceration<br>• Secondary impetigo	NA	• Dense plasma cell–rich infiltrate	NA

Galvin et al. /2016	68/F	• Pain	• Gingiva<br>• Palate<br>• Buccal mucosa	• Ulceration<br>• Erythema<br>• Desquamative gingivitis	• Skin rash<br>• Lichen Planus<br>• Arterial hypertension<br>• Hiatus hernia	• Dense plasma cell-rich infiltrate	NA
	61/F	• Pain	• Buccal mucosa<br>• Gingiva	• Edema<br>• Erythema<br>• Ulceration	• Celiac disease<br>• Hyperlipidemia<br>• Parathyroid adenoma	• Dense plasma cell-rich infiltrate<br>• Acantholysis<br>• Neutrophilic micro-abscess	NA
	69/M	• Globus pharyngeus	• Gingiva<br>• Tongue<br>• Palate<br>• Oropharynx	• Desquamative gingivitis<br>• Ulceration<br>• Tongue atrophy<br>• Papillary hyperplasia	• Ischemic heart disease<br>• Previous myocardial infarction<br>• Previuos prostate cancer<br>• Hypothyroidism<br>• Diabetes mellitus	• Dense plasma cell-rich infiltrate	NA

Mukherjee et al./ 2015	26/M	• Bleeding<br>• Swelling	• Gingiva	• Gingival hypertrophy<br>• Erythema<br>• Edema	NA	• Dense plasma cell-rich infiltrate<br>• Epithelial hyperplasia<br>• Elongated rete pegs	k:λ ratio= k>λ
Fujimura et al./ 2015	55/F	• Swelling<br>• Pain	• Lip	• Erythema<br>• Swelling	NA	• Dense plasma cell-rich infiltrate<br>• Lymphocytes	NA
Błażewicz et al./ 2015	74/F	• Dysphagia<br>• Reduction in mouth opening<br>• Swelling	• Gingiva<br>• Lip<br>• Palate<br>• Buccal mucosa	• Erythema<br>• Edema<br>• Erosion<br>• Swelling	• Arterial hypertension<br>• Diabetes mellitus type 2<br>• Coronary heart disease	• Dense plasma cell-rich infiltrate	NA
Ranganathan et al./2015	20/M	• Swelling<br>• Bleeding	• Gingiva	• Erythema	NA	• Dense plasma cell-rich infiltrate<br>• Epithelial atrophy	NA

Braga et al. /2016	40/F	• Asymptomatic	• Lip	• Warty lesion	NA	• Dense plasma cell-rich infiltrate<br>• Elongated rete ridges<br>• Exocytosis<br>• Eosinophils	NA
Khatri et al./2014	52/M	• Bleeding<br>• Burning	• Lip	• Erythema<br>• Erosion	NA	• Dense plasma cell–rich infiltrate<br>• Lymphocytes<br>• Neutrophils<br>• Macrophages	NA
da Cunha Filho et al./ 2014	58/M	• Asymptomatic	• Lip	• Ulceration<br>• Erythema	NA	• Dense plasma cell–rich infiltrate<br>• Lymphocytes<br>• Neutrophils<br>• Russell bodies	NA
Kumar et al./2015	42/M	• Pain	• Gingiva	• Exophytic and sessile lesion	NA	• Parakeratosis<br>• Epithelial hyperplasia	k:λ ratio= k>λ

		• Reduction in mouth opening<br>• Aesthetic deformities<br>• Bleeding<br>• Swelling		• Bone loss<br>• Swelling		• Dense plasma cell–rich infiltrate<br>• Russell bodies	
Gupta et al./2014	72/M	• Dysphagia<br>• Bleeding<br>• Pain<br>• Burning	• Gingiva<br>• Palate<br>• Oropharyn x<br>• Tongue<br>• Buccal mucosa	• Edema<br>• Erythema<br>• Erosion<br>• Cobblestone appearance	• Benign prostrate hyperplasia	• Dense plasma cell-rich infiltrate	Normal k:λ ratio
Madhavarajan and Tighe / 2014	63/M	• Pain<br>• Bilateral cervical lymphadenop athy	• Buccal mucosa<br>• Lip<br>• Gingiva	• Ulceration	NA	• Dense plasma cell-rich infiltrate<br>• Epithelial hyperplasia<br>• Neutrophils<br>• Macrophages	Normal k:λ ratio

Joshi and Shukla / 2015	27/M	• Bleeding<br>• Pain<br>• Swelling	• Gingiva	• Erythema<br>• Bone loss<br>• Swelling	NA	• Dense plasma cell-rich infiltrate<br>• Epithelial hyperplasia	NA
Yoshimura et al./ 2013	62/M	• Pain	• Lip	• Erosion<br>• Ulceration	NA	• Dense plasma cell-rich infiltrate<br>• Lymphocytes<br>• Dilated capillaries	NA
Makkar et al./ 2013	17/F	• Pain	• Gingiva	• Gingival hypertrophy<br>• Erythema<br>• Edema<br>• Bone loss	NA	• Epithelial hyperplasia<br>• Dense plasma cell-rich infiltrate<br>• Eosinophils	NA
Abhishek and Rashmi, /2013	16/M	• Bleeding<br>• Swelling	• Lip<br>• Gingiva	• Gingival hypertrophy<br>• Erythema	NA	• Dense plasma cell-rich infiltrate<br>• Lymphocytes	NA

				• Pseudo pockets<br>• Swelling			
Parashis et al./ 2015	32/M	• Bleeding<br>• Burning	• Gingiva	• Gingival hypertrophy<br>• Edema<br>• Erythema<br>• Bone loss	• Generalized aggressive periodontitis	• Dense plasma cell-rich infiltrate<br>• Parakeratosis<br>• Spongiosis<br>• Exocytosis<br>• Elongated rete pegs	Normal k:λ ratio
Swati et al./ 2014	21/F	• Pain<br>• Bleeding<br>• Swelling	• Gingiva	• Erythema<br>• Edema	NA	• Dense plasma cell-rich infiltrate<br>• Epithelial hyperplasia<br>• Parakeratosis<br>• Elongated rete pegs<br>• Lymphocytes<br>• Dilated capillaries	NA

	28/M	• Bleeding<br>• Swelling	• Gingiva	• Edema<br>Erythema<br>• Desquamative gingivitis<br>• Pseudo-pockets	NA	• Dense plasma cell-rich infiltrate	NA
	30/F	• Bleeding	• Gingiva	• Edema<br>• Erythema	NA	• Dense plasma cell-rich infiltrate	NA
Lamdari and Pradhan al./2012	29/F	• Bleeding<br>• Swelling	• Gingiva	• Erythema<br>• Swelling	NA	• Dense plasma cell-rich infiltrate<br>• Edema<br>• Epithelial hyperplasia<br>• Lymphocytes<br>• Dilated capillaries	NA
Bali et al./ 2012	48/F	• Bleeding<br>• Swelling	• Gingiva	• Erythema	NA	• Dense plasma cell-rich infiltrate	NA

						• Atrophy	
Janam et al./ 2012	15/F	• Swelling<br>• Bleeding	• Gingiva<br>• Lip	• Warty lesion<br>• Gingival hypertrophy<br>• Fissured lips<br>• Swelling	NA	• Dense plasma cell-rich infiltrate<br>• Epithelial hyperplasia<br>• Parakeratosis	NA
Wood et al./ 2012	70/F	• Bleeding	• Gingiva	• Gingival hypertrophy<br>• Erythema<br>• Pseudo-pockets	• Diffuse peripheral odontogenic fibroma<br>• Diabetes mellitus	• Dense plasma cell-rich infiltrate<br>• Spongiosis<br>• Exocytosis<br>• Edema	NA
Agarwal et al./ 2012	28/F	• Pain<br>• Bleeding<br>• Swelling	• Gingiva	• Gingival hypertrophy<br>• Edema<br>• Erythema<br>• Bone loss	• Allergy to clove	• Dense plasma cell-rich infiltrate<br>• Epithelial hyperplasia	NA
Shruthi et al./ 2011	57/F	• Swelling	• Alveolar mucosa	• Exophytic and sessile lesion	NA	• Dense plasma cell-rich infiltrate	NA

				• Bone loss		• Epithelial hyperplasia<br>• Parakeratosis<br>• Russell bodies	
Anil / 2007	27/F	• Swelling<br>• Bleeding	• Gingiva	• Erythema<br>• Edema	NA	• Dense plasma cell-rich infiltrate	NA
	26/M	• Bleeding	• Gingiva	• Erythema<br>• Edema	NA	• Dense plasma cell-rich infiltrate	NA
Hanami et al./ 2011	66/M	• Asymptomatic	• Lip	• Erythema<br>• Erosion	NA	• Dense plasma cell-rich infiltrate<br>• IgG+	Normal k:λ ratio
	71/M	• Asymptomatic	• Lip	• Erythema<br>• Erosion<br>• Secondary impetigo	NA	• Dense plasma cell-rich infiltrate<br>• IgG+	Normal k:λ ratio

Arduino et al./ 2011	11*/6xM, 5xF	• 11x Pain<br>• Bleeding	• 11x Gingiva	• Erythema<br>• Edema	NA	• Dense plasma<br>• cell-rich infiltrate	Normal k:λ ratio
Patil et al./ 2010	29/M	• Pain<br>• Swelling<br>• Bleeding	• Gingiva	• Erythema<br>• Pseudo pockets<br>• Bone loss<br>• Swelling	NA	• Dense plasma cell-rich infiltrate<br>• Epithelial hyperplasia<br>• Exocytosis<br>• Edema<br>• Neutrophils<br>• Lymphocytes<br>• Eosinophils	NA
Saruya et al./ 2009	49/M	• Asymptomatic	• Lip	• Erosion<br>• Secondary impetigo	NA	• Dense plasma cell-rich infiltrate<br>• Lymphocytes<br>• Dilated capillaries	NA
Choi et al./ 2009	66/F	• Bleeding	• Lip	• Erosion<br>• Secondary impetigo	NA	• Dense plasma cell-rich infiltrate	NA

	68/M	• Pain<br>• Bleeding	• Lip	• Ulceration<br>• Secondary impetigo	NA	• Dense plasma cell-rich infiltrate	NA
	65/F	• Pain<br>• Bleeding	• Lip	• Ulceration<br>• Secondary impetigo	NA	• Dense plasma cell-rich infiltrate	NA
Solomon et al. /2008	60/F	• Pain<br>• Dysphagia<br>• Reduction in mouth opening<br>• Swelling	• Gingiva<br>• Tongue<br>• Palate<br>• Lip<br>• Buccal mucosa	• Fissured tongue<br>• Erythema<br>• Erosion<br>• Swelling<br>• Small pink papules	• Fibromyalgia<br>• Sjögren syndrome<br>• Diabetes mellitus type 2<br>• Arterial hypertension<br>• Osteopenia	• Dense plasma cell-rich infiltrate<br>• IgG+	k:λ ratio = k > λ
Rawal et al./ 2008	40/M	• Pain<br>• Bleeding<br>• Swelling	• Gingiva<br>• Buccal mucosa	• Edema<br>• Erythema<br>• Ulceration<br>• Bone loss<br>• Swelling	NA	• Dense plasma cell-rich infiltrate<br>• Atrophy<br>• Edema<br>• Exocytosis	NA

Najarian et al./2008	56/M	• Bleeding<br>• Pain	• Lip	• Erosion<br>• Secondary Impetigo	• Acquired immunodeficiency syndrome<br>• Hepatitis C	• Edema<br>• Dense plasma cell-rich infiltrate<br>• Dilated capillaries	k:λ ratio = k > λ
Senol et al./2008	46/M	• Pain	• Buccal mucosa	• Erythema	NA	• Dense plasma cell-rich infiltrate<br>• Acanthosis<br>• Russell bodies<br>• Edema	Normal k:λ ratio
Farrier and Perkins/2008	53/F	• Swelling	• Lip	• Secondary Impetigo	NA	• Dense plasma cell-rich infiltrate	k:λ ratio = k > λ
Tong et al./2007	59/M	• Hoarseness<br>• Xerostomia<br>• Dysphagia<br>• Cough	• Buccal mucosa<br>• Alveolar mucosa<br>• Oropharynx	• Edema<br>• Erythema	NA	• Dense plasma cell-rich infiltrate	Normal k:λ ratio

Tseng et al./2009	55/F	• Swelling<br>• Pain<br>• Bleeding	• Lip	• Erythema<br>• Secondary impetigo<br>• Swelling	NA	• Dense plasma cell-rich infiltrate<br>• Eosinophils<br>• Macrophages	Normal k:λ ratio
Heinemann et al./ 2006	61/F	• Pain	• Tongue<br>• Lip<br>• Buccal mucosa	• Erythema<br>• Erosion/Ulceration	• Hypertonia	• Dense plasma cell-rich infiltrate<br>• Pseudo-epitheliomatous hyperplasia	NA
Yang et al./2005	56/M	• Pain	• Lip	• Erythema<br>• Erosion<br>• Secondary impetigo	• Liver cirrhosis	• Dense plasma cell-rich infiltrate<br>• Parakeratosis<br>• Edema<br>• Atrophy<br>• Dilated capillaries<br>• Neutrophils<br>• Lymphocytes	NA

	69/F	• Pain	• Lip	• Erythema<br>• Erosion<br>• Secondary impetigo	NA	• Dense plasma cell-rich infiltrate<br>• Parakeratotsis	NA
	63/F	• Pain	• Lip	• Erythema<br>• Erosion	NA	• Dense plasma cell-rich infiltrate	NA
Bharti and Smith/ 2003	42/F	• Pain<br>• Dysphagia	• Buccal mucosa<br>• Palate	• Cobblestone appearance	NA	• Dense plasma cell-rich infiltrate<br>• Neutrophils<br>• Eosinophils<br>• Lymphocytes<br>• Russell bodies	NA
Roman et al./2002	13/F	• Swelling<br>• Bleeding	• Gingiva	• Erythematous plaque	• Low levels of both serum IgA and secretory IgA	• Dense plasma cell-rich infiltrate<br>• Epithelial hyperplasia<br>• Acanthosis<br>• Atrophy	Normal k:λ ratio

Marker and Krogdahl/2002	30/M	• Pain<br>• Swelling	• Gingiva<br>• Buccal mucosa	• Erythema<br>• Ulceration<br>• Bone loss<br>• Swelling	NA	• Dense plasma cell-rich infiltrate<br>• Psoriasiform epithelial hyperplasia<br>• Exocytosis<br>• Spongiosis<br>• Dilated capillaries<br>• Neutrophils<br>• Neutrophilic micro-abscesses	Normal k:λ ratio
Kaur et al. /2001	47/M	• Swelling	• Lip	• Erythema<br>• Swelling	NA	• Dense plasma cell-rich infiltrate<br>• Acanthosis<br>• Lymphocytes	NA
Fogarty et al. /2001	27/F	• Pain<br>• Hoarseness<br>• Swelling	• Gingiva<br>• Oropharynx	• Edema<br>• Swelling	NA	• Dense plasma cell–rich infiltrate	Normal k:λ ratio

						• Pseudo-epitheliomatous hyperplasia<br>• IgG+	
Rocha et al./2004	60/F	• Bleeding<br>• Pain	• Lip	• Erythema<br>• Secondary Impetigo	• Pulmonary tuberculosis<br>• Nephrolithiasis	• Dense plasma cell–rich infiltrate<br>• Papillar epithelial hyperplasia	NA
Smith et al. /1999	59/M	• Hoarseness<br>• Swelling	• Palate<br>• Oropharynx<br>• Gingiva	• Erythematous plaques<br>• Gingival erythema	• Coeliac disease<br>• Dermatitis herpetiformis	• Epithelial hyperplasia<br>• Dense plasma cell–rich infiltrate<br>• IgG+	NA
Lee MS et al./1996	84/F	• Xerostomia<br>• Pain	• Palate<br>• Tongue<br>• Buccal mucosa<br>• Lip	• Warty lesions	• Previous carcinoma of the colon<br>• Thyrotoxicosi<br>• Atrial fibrillation	• Pseudo-epitheliomatous hyperplasia<br>• Dense plasma cell–rich infiltrate	Normal k:λ ratio

					• Coronary artery disease<br>• Previous artery cerebro-vascular accident	• Lymphocytes<br>• Eosinophils<br>• Neutrophils	
Mahler et al./1996	53/F	• Pain	• Gingiva	• Erythema	NA	•Dense plasma cell–rich infiltrate<br>•Lymphocytes<br>•IgG+ (90%)<br>•IgA+ IgM+ < 5%	Normal k:λ ratio
van de Kerkhof and van Baar/1995	80/F	• Pain	• Lip	• Erythema,<br>• Fisssured lips	• Total hip replacement<br>• Venous leg ulceration<br>• Heart failure	• Dense plasma cell–rich infiltrate<br>• Acanthosis<br>• Spongiosis<br>• Lymphocytes	NA
Tamaki et al./1994	56/M	• Pain	• Lip	• Erythema<br>• Erosion	NA	• Parakeratosis<br>• Edema	NA

						• Dense plasma cell–rich infiltrate	
	71/M	• Pain	• Lip	• Erythema<br>• Erosion	NA	• Acanthosis<br>• Edema<br>• Dense plasma cell–rich infiltrate	NA
Reed et al./ 1993	37/F	• Pain<br>• Swelling	• Gingiva	• Gingival hypertrophy<br>• Edema,<br>• Erythema<br>• Swelling	NA	• Dense plasma cell–rich infiltrate	NA
Sollecito and Greenberg /1992	61/F	• Swelling	• Gingiva<br>• Lip	• Edema<br>• Erythema	NA	• Dense plasma cell–rich infiltrate	NA
	62/F	• Pain	• Gingiva<br>• Lip	• Edema<br>• Erythema	• Hashimoto's thyroiditis<br>• Mild hypertension	• Dense plasma cell–rich infiltrate	NA

Serio et al./ 1991	37/F	• Bleeding	• Gingiva	• Erythema	NA	• Dense plasma cell–rich infiltrate	NA
Grattan et al./1992	68/M	• Swelling	• Palate<br>• Lip<br>• Oropharynx	• Papillary hyperplasia<br>• Fissured lips<br>• Swelling	• Winter bronchitis<br>• Peptic ulcer	• Epithelial hyperplasia<br>• Neutrophilic micro-abscesses<br>• Dense plasma cell–rich infiltrate<br>• IgG+	NA
	84/M	• Swelling<br>• Pain	• Palate<br>• Tongue<br>• Gingiva	• Erythema<br>• Papillary hyperplasia<br>• Erythematou plaques	NA	• Epithelial hyperplasia<br>• Atrophy<br>• Dense plasma cell–rich infiltrate<br>• Parakeratosis<br>• Neutrophilic microabscesses	NA

						• IgG+	
Timms and Sloan,/1991	70/F	• Cough<br>• Hoarseness	• Gingiva<br>• Larynx	• Erythema	• Seronegative<br>• Rheumatoid arthritis	• Dense plasma cell–rich infiltrate	NA
	32/F	• Hoarseness<br>• Sore throat<br>• Swelling	• Gingiva<br>• Larynx<br>• Buccal mucosa	• Erythema<br>• Swelling	NA	• Dense plasma cell–rich infiltrate	NA
	57/M	• Sore throat	• Gingiva<br>• Larynx	• Erythema	• Seronegative<br>• Rheumatoid arthritis	• Dense plasma cell–rich infiltrate	NA
Jones and Kennedy /1988	52/F	• Pain	• Lip	• Fissured lips	• Atopic eczema	• Parakeratosis<br>• Acanthosis<br>• Edema<br>• Elongated rete pegs<br>• Dense plasma cell–rich infiltrate	NA

Timms et al./1988	70/F	• Cough<br>• Hoarseness	• Gingiva<br>• Oropharyn<br>• Palate	• Erythema	• Rheumatoid arthritis	• Dense plasma cell–rich infiltrate<br>• Pseudo-epitheliomatous hyperplasia<br>• Lymphocytes<br>• Macrophages<br>• IgG+ IgA+IgM+	Normal k:λ ratio
White et al. /1986	47/F	• Pain<br>• Sore throat<br>• Globus pharyngeus	• Buccal mucosa<br>• Oropharyn<br>• Lip<br>• Tongue	• Erythema<br>• Fissured lip	• Psoriasis	• Acanthosis<br>• Atrophy<br>• Edema<br>• Dense plasma cell–rich infiltate	Nrmal k:λ ratio
Lubow et al./1984	36/M	• Pain<br>• Bleeding	• Gingiva	• Ulceration	• Psoriasis	• Psoriasiform epithelial hyperplasia<br>• Atrophy	NA

						• Dense plasma cell–rich infiltrate	
Palmer and Eveson/1981	29/F	• Swelling	• Gingiva	• Erythema	• Asthma	• Psoriasiform epithelial hyperplasia<br>• Elongated rete pegs<br>• Atrophy<br>• Spongiosis<br>• Dense plasma cell–rich infiltrate<br>• Neutrophils<br>• Lymphocytes	NA
	52/M	• Asymptomatic	• Gingiva	• Erythema	• Psoriasis<br>• Paget's disease	• Psoriasiform epithelial hyperplasia<br>• Elongated rete pegs<br>• Atrophy<br>• Spongiosis	NA

						• Dense plasma cell–rich infiltrate<br>• Neutrophils<br>• Lymphocytes	
Silverman and Lozada /1977	42,5*/12xF, 4xM	• x9 Bleeding<br>• x16 Pain	• x1 Buccal mucosa<br>• x1 Floor of the mouth<br>• x16 Gingiva<br>• x15 Tongue<br>• x16 Lip	• x16 erythema<br>• x16 Angular Cheilitis	• x2 Cardiovascular disease<br>• x1 Polycythemia vera,<br>• x1 Arthritis<br>• x1 Low hemoglobin<br>• x1 Allergies	• Dense plasma cell–rich infiltrate	NA
Baughman et al./ 1974	67/F	• Pain	• Lip	• Edema	• Mild degenerative arthritis<br>• Hypertension	• Acanthosis<br>• Elongated rete pegs<br>• Spongiosis<br>• Exocytosis<br>• Dense plasma cell–rich infiltrate	NA

Kerr et al./1971	59/F	• Burning<br>• Swelling	• Gingiva<br>• Lip	• Angular cheilitis<br>• Swelling	• Carcinoma of the endometrium	• Papillar epithelial hyperplasia<br>• Dense plasma cell–rich infiltrate<br>• Dilated capillaries<br>• Mast cells	NA
	27/F	• Pain<br>• Dysphagia	• Gingiva<br>• Palate<br>• Lip<br>• Buccal mucosa<br>• Tongue	• Angular cheilitis<br>• Secondary impetigo<br>• Edema<br>• Erythema	NA	• Papillar epithelial hyperplasia<br>• Dense plasma cell–rich infiltrate<br>• Dilated capillaries<br>• Mast cells	NA
	11/F	• Pain<br>• Swelling<br>• Bleeding	• Gingiva<br>• Lip<br>• Oropharyn x	• Angular cheilitis<br>• Secondary impetigo	• Frequent upper respiratory infections<br>• Ashma	• Papillar epithelial hyperplasia	NA

			• Tongue	• Fissured tongue and lip<br>• Edema<br>• Erythema<br>• Gingival<br>• Hyperplasia<br>• Swelling		• Dense plasma cell–rich infiltrate<br>• Dilated capillaries<br>• Mast cells	
	28/F	• Pain<br>• Bleeding<br>• Swelling	• Gingiva<br>• Lip<br>• Oropharynx	• Angular cheilitis<br>• Fissured lip<br>• Swelling<br>• Edema<br>• Erythema	• Angioneurotic edema	• Papillar epithelial hyperplasia<br>• Dense plasma cell–rich infiltrate<br>• Dilated capillaries<br>• Mast cells	NA
	27/M	• Pain<br>• Bleeding<br>• Swelling	• Gingiva<br>• Lip<br>• Tongue<br>• Buccal mucosa	• Edema<br>• Angular cheilitis<br>• Secondary impetigo	NA	• Papillar epithelial hyperplasia	NA

				• Erythema<br>• Swelling		• Dense plasma cell–rich infiltrate<br>• Dilated capillaries<br>• Mast cells	
	18/F	• Pain	• Gingiva<br>• Lip<br>• Tongue	• Erythema<br>• Angular cheilitis<br>• Fissured lip<br>• Tongue atrophy	• Allergy	• Papillar epithelial hyperplasia<br>• Dense plasma cell–rich infiltrate<br>• Dilated capillaries<br>• Mast cells	NA
	24/F	• Pain<br>• Dysphagia	• Gingiva<br>• Lip<br>• Tongue<br>• Buccal mucosa	• Angular cheilitis<br>• Fissured lip<br>• Fissured tongue<br>• Gingival Hyperplasia	NA	• Papilalr epithelial hyperplasia<br>• Dense plasma cell–rich infiltrate	NA

				• Erythema		• Dilated capillaries<br>• Mast cells	
	21/M	• Pain<br>• Bleeding	• Gingiva<br>• Tongue<br>• Lip	• Angular cheilitis<br>• Fissured lip<br>• Fissured tongue<br>• Erythema	NA	• Papilar epithelial hyperplasia<br>• Dense plasma cell–rich infiltrate<br>• Dilated capillaries<br>• Mast cells	NA
Owings/ 1969	26/F	• Pain<br>• Bleeding<br>• Swelling	• Gingiva<br>• Tongue<br>• Lip	• Edema<br>• Erythema<br>• Angular cheilitis<br>• Fissured tongue<br>• Swelling	NA	• Epithelial hyperplasia<br>• Dense plasma cell–rich infiltrate<br>• Exocytosis	NA
	47/F	• Pain	• Gingiva<br>• Tongue<br>• Lip	• Erythema<br>• Angular Cheilitis	• Arthritis<br>• Thyroid disease	• Dense plasma cell–rich infiltrate	NA

						• Edema<br>• Atrophy	
	44/F	• Pain<br>• Dysphagia	• Gingiva<br>• Tongue<br>• Lip	• Edema<br>• Erythema<br>• Fissured tongue<br>• Angular Cheilitis	• Ovarian cyst	• Epithelial hyperplasia<br>• Parakeratosis<br>• Elongated rete pegs<br>• Dense plasma cell–rich infiltrate<br>• Mast cells<br>• Lymphocytes	NA
Poswillo /1968	37/F	• Swelling	• Gingiva	• Hyperplasia	NA	• Acanthosis<br>• Dense plasma cell–rich infiltrate	NA
	39/F	• Asymptomatic	• Gingiva	• Gingival hypertrophy	NA	• Acanthosis<br>• Dense plasma cell–rich infiltrate	NA
	21/F	• Asymptomatic	• Gingiva	• Hyperplasia	• Down syndrome	• Acanthosis	NA

			• Buccal mucosa	• Gingival hypertrophy		• Dense plasma cell–rich infiltrate	
Lightstone et al. /1960	45/M	• Xerostomia<br>• Asthenia<br>• Bleeding<br>• Pain	• Lip<br>• Oropharynx	• Erosion/Ulceration	• Multiple myeloma	• Edema<br>• Dense plasma cell–rich infiltrate<br>• Lymphocytes	NA
Micucci et al./2021	57/M	• Bleeding<br>• Pain	• Palate	• Erythema/Ulceration	• Dermatitis<br>• Eczema	• Dense plasma cell–rich infiltrate	Normal k:λ ratio
Cohen et al./2021	80/M	• Bleeding<br>• Pain<br>• Burning	• Lip	• Erythema/Ulceration<br>• Secondary impetigo<br>• Cheilitis	• Myelodysplastic Sindrome<br>• Anemia	• Dense plasma cell–rich infiltrate<br>• Lymphocytes	Normal k:λ ratio
Tailor et al./2021	47/M	• Burning<br>• Bleeding<br>• Dysphonia<br>• Dysphagia	• Lip<br>• Buccal mucosa<br>• Palate<br>• Gingiva<br>• Tongue	• Erythema/Ulceration<br>• Desquamative gingivitis<br>• Cobbleston appearance	• Schizophrenia	• Dense plasma cell–rich infiltrate	Normal k:λ ratio

				• Fissured tongue<br>• Secondary impetigo			
	39/M	• Pain	• Palate<br>• Gingiva	• Erythema/Ulceration	NA	• Edema<br>• Dense plasma cell–rich infiltrate	NA
Vishnu et al./2020	13/F	• Swelling<br>• Bleeding	• Gingiva	• Erythema<br>• Edema<br>• Pseudo-pockets<br>• Gingival hypertrophy	NA	• Epithelial hyperplasia<br>• Dense plasma cell–rich infiltrate<br>• Dilated capillaries<br>• Limphocytes<br>• Fibrosis	NA
Ketkar et al./2021	24/F	• Swelling<br>• Bleeding	• Gingiva	• Desquamative gingivitis<br>• Pseudo-pockets	• Asymptomatic patchy skin lesions	• Dense plasma cell–rich infiltrate	NA

				• Swelling		• Dilated capillaries<br>• Parakeratosis	
Genet et al.,/2021	61/F	• Swelling<br>• Bleeding<br>• Pain	• Gingiva	• Edema<br>• Swelling	• IgG kappa multiple myeloma (MM) in complete remission<br>• Hypothyroidism<br>• Primary Sjögren's syndrome<br>• Depression	• Dense plasma cell–rich infiltrate	Normal k:λ ratio
Lu et al., /2020	15/M	• Swelling<br>• Bleeding<br>• Pain	• Gingiva	• Exophytic, pedunculate d, lesion	NA	• Epithelial hyperplasia<br>• Dense plasma cell–rich infiltrate<br>• Russel bodies<br>• Dilated capillaries	Normal k:λ ratio

Konidena et.,/2014	28/M	• Swelling<br>• Bleeding	• Gingiva	• Gingival hypertrophy	• Muscular dystrophy type II	• Dense plasma cell–rich infiltrate	NA
Shivalingu et al., /2016	12/F	• Swelling<br>• Bleeding<br>• Burning	• Gingiva	• Erythema<br>• Edema<br>• Desquamative gingivitis	NA	• Dense plasma cell–rich infiltrate	NA
Negi et al.,/2019	12/M	• Swelling<br>• Bleeding	• Gingiva	• Erythema<br>• Edema<br>• Pseudo-pockets	NA	• Epithelial hyperplasia<br>• Parakeratosis	Normal k:λ ratio
Chauhan et al.,/2019	18/M	• Swelling<br>• Bleeding	• Gingiva<br>• Lip	• Swelling<br>• Edema<br>• Gingival hypertrophy<br>• Pseudo-pockets	NA	• Dense plasma cell–rich infiltrate<br>• Parakeratosis<br>• Elongated rete pegs	Normal k:λ ratio

						• Lymphocytes	
Antonelli et al., /2020	14/F	• Pain<br>• Burning<br>• Dysgeusia<br>• Hoarseness<br>• Sore throat	• Tongue<br>• Gingiva	• Edema<br>• Erythema<br>• Ulceration	NA	• Dense plasma cell–rich infiltrate	k:λ ratio= k> λ
Gasparro et al./2019	78/F	• Pain	• Buccal mucosa	• Erythema<br>• Erosion<br>• Ulceration	• Hypertension	• Dense plasma cell–rich infiltrate	NA
Macleod et al., /1989	47 M	• Bleeding	• Gingiva	• Hyperplasia<br>• Erythema<br>• Desquamati on	• Hayfever	• Edema<br>• Dense plasma cell–rich infiltrate<br>• Dilated capillaries<br>• Neutrophilic micro-abscess	k:λ ratio= k> λ
